# Association between the serum alpha-1-acid glycoprotein concentrations and depression in US adult women: a cross-sectional study

**DOI:** 10.1186/s12888-025-06934-w

**Published:** 2025-05-15

**Authors:** Yuanyuan Zhong, Chunyue Fang, Tianliang Yao, Hongjin Wang, Min Jiang

**Affiliations:** 1 Department of Cardio-Thoracic Surgery, Longyan First Affiliated Hospital of Fujian Medical University, Longyan, China; 2 The First Affiliated Hospital , College of Clinical Medicine of Henan University of Science and Technology, Luoyang, China; 3 Department of Pharmacy, The Third People’s Hospital of Yunnan Province, Kunming, Yunnan, 650011 China; 4 College of Pharmacy, Dali University, Dali, Yunnan China; 5 Department of Control Science and Engineering, College of Electronic and Information Engineering, Tongji University, No. 1239, Siping Road, Shanghai, 200092 China

**Keywords:** Alpha-1-acid glycoprotein levels, Depression, PHQ-9 scores

## Abstract

**Background:**

Emerging evidence has demonstrated a positive association of inflammation with depression. As an acute-phase reactant predominantly synthesized in hepatocytes, alpha-1-acid glycoprotein (AGP) serves as a sensitive biomarker of inflammation. However, there is a limited study to explore the relationship between AGP and depression. Currently, the association of AGP with depression is controversial.

**Methods:**

This study utilized data from the National Health and Nutrition Examination Survey (NHANES) collected between 2021 and 2023. The Patient Health Questionnaire-9 (PHQ-9) was employed to assess depressive symptoms, with a score ≥ 10 indicating clinically relevant depression. We utilized weighted multivariate logistic regression for depression outcomes, weighted linear regression for continuous PHQ-9 scores, and restricted cubic splines (RCS) to examine potential nonlinear relationships between AGP and depression. To evaluate the robustness of these associations, we conducted comprehensive subgroup analyses with interaction tests and multiple sensitivity analyses.

**Results:**

Serum AGP concentrations exhibited a significant positive association with depression among U.S. adult women, demonstrating a linear dose-response relationship. In the fully adjusted model, each ln-unit increase in AGP concentrations was associated with a 1.13-fold higher odds ratio of depression (OR: 2.13, 95% CI: 1.26–3.64) and a 1.47-point elevation in PHQ-9 values (β: 1.47, 95% CI: 0.37–2.56). Moreover, participants in the highest AGP quartile had a 0.72-fold increased odds ratio of depression (OR: 1.72, 95% CI: 1.03–2.87) and a 1.32-point higher PHQ-9 score (β:1.32, 95% CI: 0.31–2.34) compared to those in the lowest quartile. This positive association remained consistent across multiple subgroup analyses.

**Conclusions:**

Serum AGP concentrations demonstrated a significant positive linear association with depressive symptoms among nationally representative samples of U.S. adult women.

**Supplementary Information:**

The online version contains supplementary material available at 10.1186/s12888-025-06934-w.

## Introduction

Depression represents a severe psychiatric condition characterized by persistent depressed mood, marked anhedonia, and associated cognitive deficits, behavioral alterations, and autonomic dysregulation, collectively contributing to significant somatic health consequences [1–3]. The prevalence of depression has risen markedly in recent years due to the escalating pressures of modern lifestyles, occupations, and academics. Globally, an estimated 280 million people were diagnosed with depression, representing approximately 3.8% of the population, with adults accounting for 5.0% of cases [4, 5]. In addition, over 700,000 annual suicide deaths are attributed to depression, imposing substantial health and socioeconomic burdens on individuals, families, and societies [6, 7]. Emerging evidence identifies some risk factors, such as sedentary behavior, socioeconomic disadvantage, poor dietary patterns, sleep deprivation, and comorbid conditions, were linked with the prevalence of depression [8, 9]. Additionally, dysregulated inflammatory responses, oxidative stress, and disruptions in glucose and lipid metabolism as key mechanistic drivers of depression onset and progression [10–15]. Nevertheless, the precise etiology and underlying molecular pathways remain incompletely elucidated.

Alpha-1-acid glycoprotein (AGP) is an acute-phase reactant predominantly produced in hepatocytes with additional synthesis occurring in pulmonary alveolar type II cells [16, 17]. Following its production, AGP is rapidly secreted into the systemic circulation through hepatic sinusoidal endothelium and subsequently diffuses across extravascular compartments, including cerebrospinal fluid, gastrointestinal secretions, and urine [18, 19]. During inflammatory states characterized by redox imbalance, the resultant accumulation of oxidative byproducts and proinflammatory mediators triggers a marked upregulation of AGP synthesis [20, 21]. As a sensitive inflammatory marker, it is significantly associated with the pathogenesis of various metabolic and inflammatory conditions, including nonalcoholic fatty liver disease, acute kidney injury, cardiovascular disorders, and obesity [22–26]. In addition, emerging evidence has demonstrated that elevated serum AGP concentrations are associated with increased depression risk. A cross-sectional descriptive and comparative study involving 65 clinically depressed patients and 30 healthy controls reported significantly higher AGP concentrations in the patient group [27]. However, these findings have not been consistently replicated across studies, with some reporting null associations [28]. These discrepancies may stem from methodological limitations, including small sample sizes, heterogeneous study designs, and inadequate control for potential confounders such as inflammatory comorbidities. Given these inconsistencies in the current studies, a large-scale epidemiological investigation is warranted to elucidate the relationship of serum AGP concentrations with depression.

## Methods

### Study design and participants

This cross-sectional study employed data from the 2021–2023 NHANES cycle, a population-based surveillance system utilizing a complex, probability-based sampling design to monitor health status and dietary patterns across the United States [29]. Implemented by the National Center for Health Statistics (a division of the Centers for Disease Control and Prevention), the study incorporates both self-reported measures collected through household interviews and objective biometric assessments performed at specialized field clinics [30]. The investigation received National Center for Health Statistics Research Ethics Review Board approval (Protocol #2021-05), with documented consent obtained from all enrolled individuals before their participation [31].

**Fig. 1:**
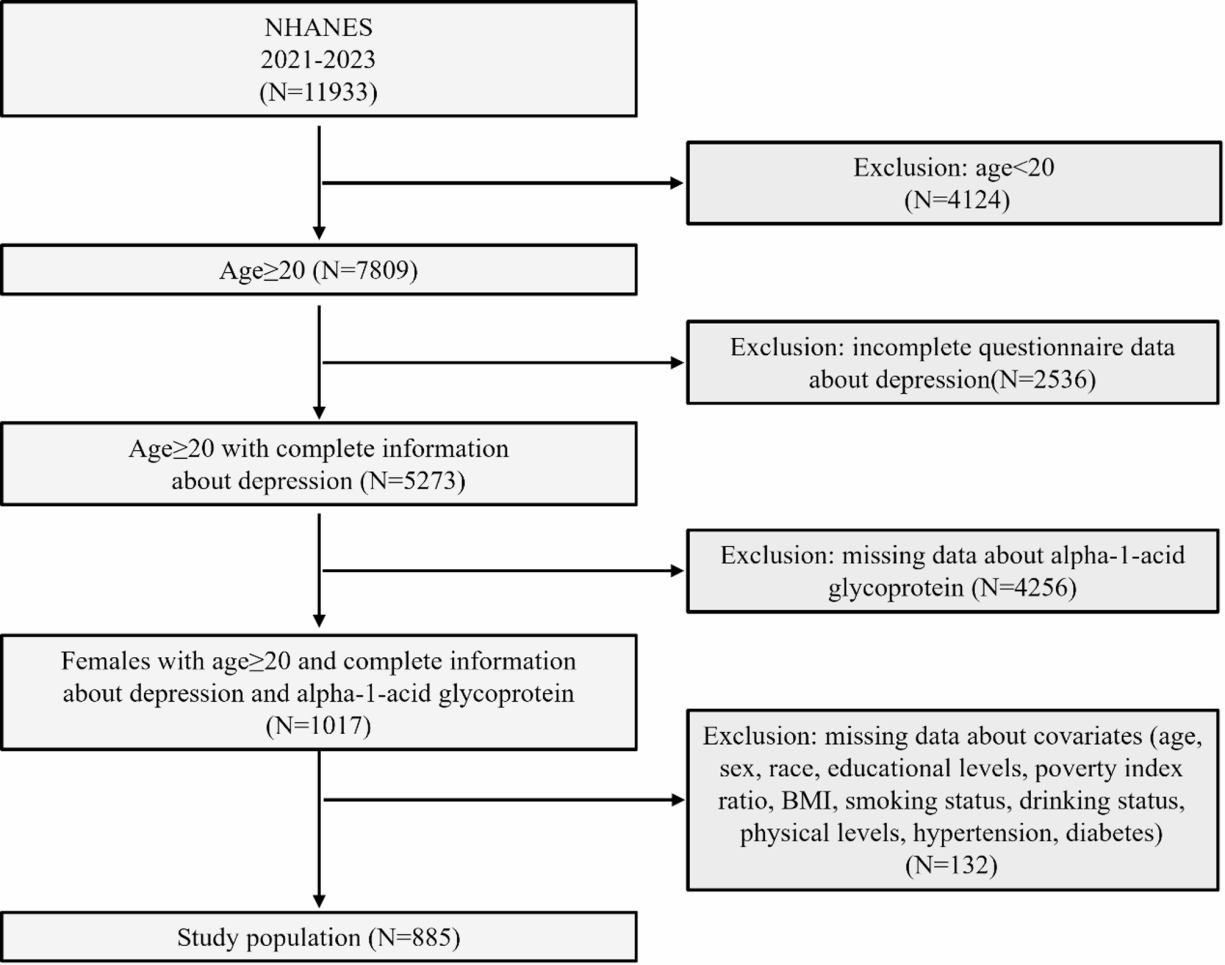
Flow chart of participants in inclusion and exclusion criteria

Our analytical samples were derived from an initial pool of 11,933 individuals surveyed during the 2021–2023 study period. Through sequential exclusion criteria application, we removed: (1) 4124 participants aged < 20 years; (2) 2536 cases with incomplete depression screening data; (3) 4256 subjects lacking AGP measurements; and (4) 132 individuals with incomplete covariate information. The final analytical sample comprised 885 eligible participants, representing a weighted national population estimate of 6.31 million US adults. Figure 1 illustrated the complete participant selection flowchart.

### Measurement of the AGP

Serum AGP quantification was conducted using the Tina-quant Roche AGP assay based on the principle of immunological agglutination. The quantitative methodology employed an immunoturbidimetric detection system based on antigen-antibody interaction. Specifically, AGP molecules formed immunocomplexes with corresponding antibodies, resulting in light-scattering aggregates. These aggregates were quantified spectrophotometrically by measuring the increase in solution turbidity proportional to AGP concentration [32].

### Depression definitions

Depressive symptoms were evaluated using the PHQ-9 [33, 34], a validated screening tool derived from the Diagnostic and Statistical Manual of Mental Disorders, Fourth Edition (DSM-IV) criteria [35]. Each of the nine items, corresponding directly to the core DSM-IV diagnostic criteria for major depressive disorder, was scored on a 4-point Likert scale (0 = not at all to 3 = nearly every day), based on symptom frequency during the preceding two-week period [36]. Total scores ranged from 0 to 27, with well-established cut-off scores demonstrating good reliability (Cronbach’s α = 0.89) and validity. Consistent with standard clinical practice, participants scoring ≥ 10 were classified as having clinically significant depression, a threshold demonstrating 88% sensitivity for major depressive disorder diagnosis [37].

### Covariates

This study incorporated comprehensive demographic, socioeconomic, and clinical covariates. Age stratification comprised young (20–34 years) and middle-aged (35–49 years) adults. Racial classification included Non-Hispanic White, Non-Hispanic Black, Mexican American, and other races. Educational attainment was trichotomized (less than high school, high school graduate, college or above). Household income levels were determined by poverty-income ratio thresholds (< 1.0, 1.0-2.9, ≥ 3.0). Body mass index (BMI) measures follow WHO classifications. Alcohol/tobacco use history captured current, former, and never users. Physical activity intensity was graded as vigorous, moderate, or low based on metabolic equivalents. Hypertension criteria incorporated JNC-8 guidelines (≥ 130/80 mmHg, antihypertensive use, or clinical diagnosis) [38]. Diabetes mellitus was defined per ADA standards (fasting glucose ≥ 126 mg/dL, HbA1c ≥ 6.5%, hypoglycemic agents, or physician diagnosis) [39]. Both chronic conditions were analyzed as dichotomous variables (yes/ no).

### Statistical analysis

In this study, To make the data nationally representative, we used “SDMVPSU” and “SDMVSTRA” to conduct NHANES’s survey design, and “WTINT2YR” to provide weight for the data. Participant characteristics were compared using Student’s t-tests for continuous variables and Pearson’s χ² tests for categorical variables, with appropriate normality and variance assumptions verified. Initial analysis revealed a non-normal distribution of serum AGP concentrations (Shapiro-Wilk test, *P* > 0.05; Figure S1A). Therefore, to reduce this skewness, we applied natural logarithmic transformation, which successfully normalized the distribution (Shapiro-Wilk test, *P* < 0.05; Figure S1B). The ln-transformed values were used in subsequent statistical analysis. AGP was analyzed both as a continuous variable and as quartile-based categories: Q1 (0.10–0.78 mg/mL), Q2 (0.79–1.45 mg/mL), Q3 (1.46–1.89 mg/mL), and Q4 (1.90–2.76 mg/mL). The association between logarithmically transformed AGP concentrations and both depressive status (dichotomous outcome) and symptom severity (continuous PHQ-9 scores) was examined through three complementary analytical approaches: (1) multivariable-adjusted logistic regression for categorical depression classification, (2) linear regression modeling for dimensional symptom assessment, and (3) restricted cubic spline (RCS) regression to evaluate potential nonlinear dose-response relationships. All analyses incorporated sampling weights to ensure population representativeness. In this study, model selection for RCS was based on minimizing the Akaike Information Criterion (AIC), with optimal performance observed at 3 knots (AIC = 2451 for depression status; AIC = 2517 for PHQ-9 scores; Tables S1-S2). To evaluate the stability of the observed relationship of AGP concentrations with depressive symptoms, subgroup analyses, and sensitive analyses were conducted. On the one hand, missing covariate values were imputed using multiple interpolation techniques, including predictive mean matching methods, to preserve dataset completeness while minimizing imputation bias, and further reducing the effect of these missing data on the final result. On the other hand, to assess the association between serum AGP concentrations and depressive symptoms in the unweighted data, we conducted multivariate logistical and linear models to validate. All statistical computations were executed using R statistical software (v4.3.2). A two-tailed *P*-value of 0.05 served as the threshold for determining statistical significance throughout our analytical procedures.

## Result

### Baseline characteristics of the participants

Table 1 summarized the demographic and clinical characteristics of the study, comprising 885 female participants aged 20 years or older. 183 (20.68%) of these participants had depression and the other 702 (79.32%) did not. Significant between depression and non-depression group differences (*P* < 0.05) were observed across multiple parameters, including socioeconomic status (PIR), anthropometric measures (BMI), health behaviors (tobacco use and physical activity), cardiometabolic comorbidities (hypertension and diabetes status), AGP concentrations and depressive symptom severity (PHQ-9 scores).

**Table 1:** Baseline characteristics of the participants

Variable	Total (*n* = 885), n (%) or mean (se)	Depression (*n* = 183), n (%) or mean (se)	None-depression (*n* = 702), n (%) or mean (se)	*P*-value
Age	36.21 (8.21)	34.75 (8.32)	36.53 (8.13)	0.325
Race Non-Hispanic White Non-Hispanic Black Mexican American Other races	481 (54.35) 109 (12.31) 80 (9.05) 215 (24.29)	112 (61.22) 23 (12.56) 17 (9.28) 31 (16.94)	369 (52.56) 86 (12.25) 63 (8.97) 184 (26.22)	0.702
Educational attainment Less than High-school High school College or above	72 (8.13) 128 (14.46) 685 (77.41)	24 (13.11) 37 (20.22) 122 (66.67)	48 (6.84) 91 (12.96) 563 (80.20)	0.214
PIR PIR < 1 1 ≤ PIR < 3 PIR>3	163 (18.42) 343 (38.76) 379 (42.82)	51 (27.87) 102 (55.74) 30 (16.39)	112 (15.95) 241 (34.33) 349 (49.72)	0.003
BMI Underweight (< 18.5) Normal weight (18.5–24.9) Overweight (25-29.9) Obesity (≥ 30)	26 (2.94) 242 (27.34) 263 (29.72) 354 (40.00)	16 (8.74) 42 (22.95) 54 (29.51) 71 (38.80)	10 (1.42) 200 (28.49) 209 (29.77) 283 (40.32)	0.002
Drinking status Never drinker Former drinker Current drinker	74 (8.36) 68 (7.68) 743 (83.96)	25 (13.66) 20 (10.93) 138 (75.41)	49 (6.98) 48 (6.85) 605 (86.17)	0.523
Smoking status Never smoker Former smoker Current smoker	598 (67.57) 165 (18.64) 122 (13.79)	104 (56.83) 37 (20.21) 42 (22.96)	494 (70.37) 128 (18.23) 80 (11.40)	0.009
Physical activities Vigorous Middle Other	476 (53.78) 277 (31.29) 132 (14.93)	79 (43.16) 68 (37.17) 36 (19.67)	397 (56.56) 209 (29.77) 96 (13.67)	0.005
Hypertension Yes No	265 (29.94) 620 (70.06)	82 (44.81) 101 (55.19)	183 (26.07) 519 (73.93)	0.001
Diabetes Yes No	83 (9.38) 802 (90.62)	34 (18.58) 149 (81.42)	49 (6.98) 653 (93.02)	0.002
AGP (g/L)	0.81 (0.32)	0.89 (0.21)	0.76 (0.34)	0.005
PHQ-9 score	5.84 (5.21)	14.43 (4.20)	3.21 (2.81)	0.004

**Notes**: Categorical data were expressed as proportions (%) and analyzed using Pearson’s χ² test for between-group comparisons. Normally distributed continuous variables were reported as mean ± standard error (SE) and evaluated with Student’s t-test

### Associations between serum AGP concentrations and depression

**Fig. 2:**
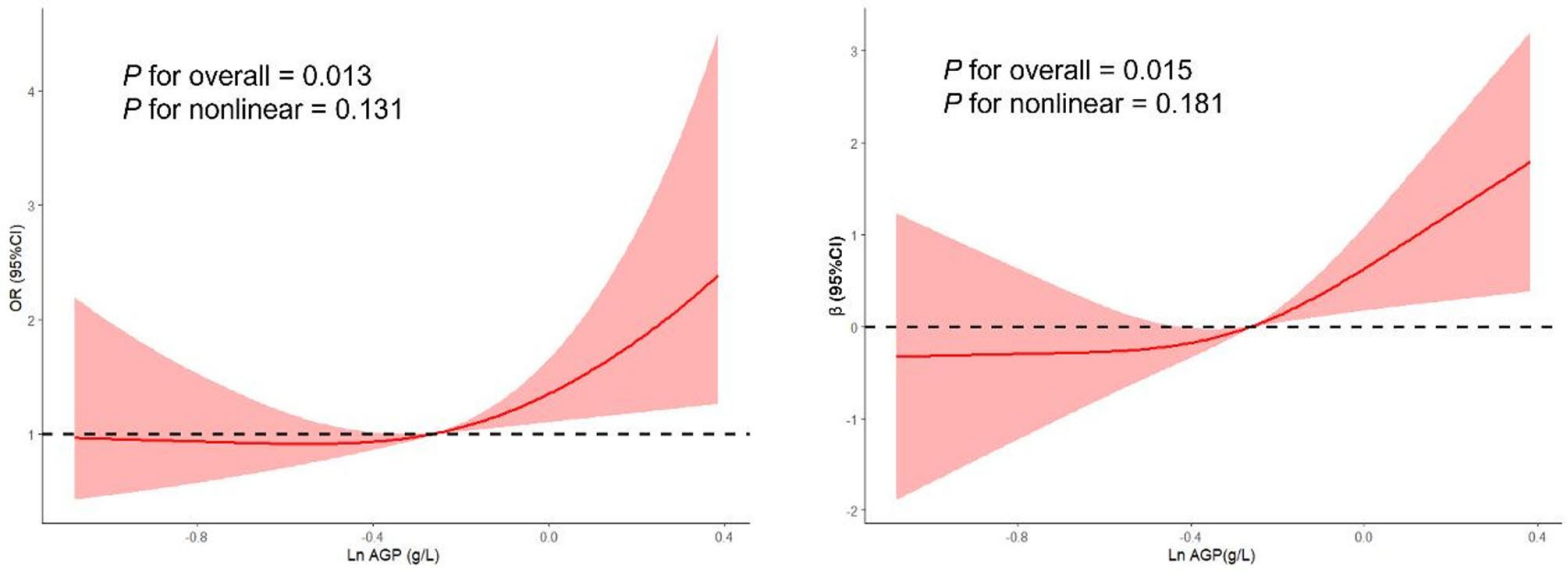
The dose-response relationships between natural logarithm-transformed AGP concentrations and both depression status and continuous PHQ-9 values

Table 2 demonstrates a significant positive correlation between AGP concentrations and depressive symptoms and their severity (PHQ-9 scores). Multivariable-adjusted logistic regression analyses demonstrated a dose-dependent relationship between natural log-transformed AGP concentrations and depression. Progressive adjustment for covariates attenuated but maintained the significant association, with adjusted odds ratios of 2.87 (95%CI: 1.69–4.86; *P* < 0.001) in model 1, 2.41 (95%CI: 1.43–4.23; *P* < 0.001) in model 2, and 2.13 (95%CI: 1.26–3.64; *P* < 0.001) in model 3, corresponding to 187%, 141%, and 113% relative odds ratio increases per ln-unit AGP increment, respectively. Stratified by AGP quartiles, participants in the highest concentration quartile exhibited significantly greater depression odds ratio versus the lowest quartile referent group. The magnitude of association progressively attenuated with sequential covariate adjustment: OR = 2.36 (95%CI: 1.46–3.82, *P* < 0.001) in model 1, OR = 1.95 (95%CI: 1.18–3.23, *P* = 0.008) in model 2, and OR = 1.72 (1.03–2.87, *P* = 0.032) in model 3, corresponding to 136%, 95%, and 72% relative odds ratio elevations, respectively. In addition, our results of linear trend analysis revealed an increased linear trend of an odds ratio of depressive symptoms with elevated AGP concentration in three models (all *P* < 0.05). Notably, serum AGP concentrations demonstrated a consistent positive correlation with PHQ-9 scores in multivariable-adjusted linear regression analyses, mirroring the categorical depression outcomes. Additionally, there was also an increased trend of the AGP concentrations with PHQ-9 values in three models (all *P* < 0.05). In addition, the results of Fig. 2 also showed that a linear dose-response relationship between ln-transform AGP, depression (*P* - overall = 0.013, *P -* nonlinear = 0.131), and the PHQ-9 scores (*P*- overall = 0.015, *P* - nonlinear = 0.181) was observed in RCS models after adjusting all covariates, respectively. In these dose-response curves, the odds ratio of depression was increased with elevated serum AGP concentrations.

**Table 2:** Association between ln-serum AGP concentrations and depression and PHQ-9 scores

	OR (95% CI)	*P*-value	β (95% CI)	*P*-value
Model 1				
Continuous	2.87 (1.69, 4.86)	< 0.001	2.23 (1.15, 3.31)	< 0.001
Q1	Reference (1.00)		Reference (0.00)	
Q2	1.34 (0.78, 2.37)	0.212	0.72 (-0.26, 1.72)	0.151
Q3	1.59 (0.96, 2.62)	0.067	1.25 (0.26, 2.25)	0.014
Q4	2.36 (1.46, 3.82)	< 0.001	2.05 (1.05, 3.05)	< 0.001
*P* for trend	0.001		0.001	
Model 2				
Continuous	2.41 (1.43, 4.23)	< 0.001	1.84 (0.76, 2.92)	< 0.001
Q1	Reference (1.00)		Reference (0.00)	
Q2	1.27 (0.76, 2.31)	0.198	0.61 (-0.35, 1.58)	0.212
Q3	1.49 (0.89, 2.49)	0.123	1.14 (0.16, 2.12)	0.022
Q4	1.95 (1.18, 3.23)	0.008	1.65 (0.66, 2.65)	0.001
*P* for trend	0.003		0.002	
Model 3				
Continuous	2.13 (1.26, 3.64)	< 0.001	1.47 (0.37, 2.56)	0.008
Q1	Reference (1.00)		Reference (0.00)	
Q2	1.21 (0.70, 2.09)	0.454	0.47 (-0.49,1.44)	0.339
Q3	1.36 (0.81, 2.28)	0.242	0.91 (-0.06, 1.90)	0.066
Q4	1.72 (1.03, 2.87)	0.032	1.32 (0.31, 2.34)	0.010
*P* for trend	0.028		0.009	

**Abbreviations**: Q1–Q4: first to fourth quartiles; Ref: reference group. **Model**: Model 1 (unadjusted); Model 2 (demographic-adjusted: age, race, educational attainment, and PIR); Model 3 (fully adjusted: including all covariates)

### Subgroup analyses

In this study, subgroup analyses were performed to evaluate the consistency of the observed association between depressive symptom severity and AGP concentrations across clinically relevant demographic and metabolic subgroups. Table 3 and Table S3 showed significant positive correlations between serum AGP concentrations and depressive symptoms across specified demographic strata (all *P* < 0.05), such as participants aged 20–34 years (OR = 2.34, 95%CI: 1.06–5.14), Non-Hispanic White (OR = 2.66, 95%CI: 1.25–5.67), those attaining the education levels of a college degree or higher (OR = 2.11, 95%CI: 1.10–4.03), those with PIR from 1 to 3 (OR = 5.71, 95%CI: 2.43–13.42), those with current drinker (OR = 2.94, 95% CI: 1.55–5.57), those with non-smoker (OR = 2.23, 95% CI: 1.12–4.14), those with vigorous exercise levels (OR = 2.89, 95%CI: 1.25–6.68), suggesting the observed association may demonstrate generalizability across diverse demographic strata. Furthermore, interaction analyses revealed no statistically significant effect modification by any subgroup characteristics (all interaction *P*-values > 0.05).

**Table 3:** Subgroup analyses of the association between serum AGP concentrations and depressive disorder across subgroups

Subgroups	OR (95% CI)	*P*-value	*P* for interaction
Age 20–34 years 35–49 years	2.34 (1.06, 5.14) 1.91 (0.82, 4.41)	0.034 0.128	0.695
Ethnicity Non-Hispanic White Non-Hispanic Black Mexican American Other races	2.66 (1.25, 5.67) 0.51 (0.09, 2.92) 2.22 (0.42, 9.00) 1.39 (0.44, 4.31)	0.011 0.454 0.183 0.566	0.423
Educational levels Less than High-school High school College or above	1.12 (0.11, 10.65) 2.78 (0.63, 12.23) 2.11 (1.10, 4.03)	0.924 0.174 0.023	0.962
Poverty index ratio PIR < 1 1 ≤ PIR < 3 PIR **>** 3	0.41 (0.12, 1.39) 5.71 (2.43, 13.42) 1.50 (0.51, 4.41)	0.152 < 0.001 0.457	0.324
BMI Underweight Normal weight Overweight Obesity	1.49 (0.25, 7.60) 2.10 (0.66, 6.58) 0.63 (0.18, 2.25) 2.32 (0.78, 6.83)	0.291 0.203 0.484 0.126	0.861
Drinking status Never drinker Former drinker Current drinker	1.07 (0.14, 8.09) 0.61 (0.08, 4.58) 2.94 (1.55, 5.57)	0.950 0.636 < 0.001	0.643
Smoking status Never smoker Former smoker Current smoker	2.23 (1.12, 4.14) 0.89 (0.32, 3.53) 3.37 (0.79, 14.21)	0.013 0.867 0.127	0.523
Physical activities Vigorous Middle Other	2.89 (1.25, 6.68) 1.93 (0.75, 4.95) 1.93 (0.43, 8.66)	0.012 0.170 0.607	0.092
Hypertension Yes No	2.32 (0.79, 5.87) 1.87 (0.89, 3.62)	0.241 0.097	0.864
Diabetes Yes No	2.74 (0.62, 13.32) 1.97 (0.98, 3.89)	0.153 0.068	0.753

**Notes**: All covariates were adjusted in the model

### Sensitivity analyses

To assess the robustness of our findings, we performed comprehensive sensitivity analyses evaluating the AGP-depression relationship through multiple model specifications. These included analyses addressing potential bias from covariate missingness that could influence the observed associations with both depression status and continuous symptom severity scores. Multiple imputation techniques were employed to address missing covariate data, which were interpolated by the predictive mean matching method after using the “MICE” package in R. The multiple imputation sensitivity analysis confirmed the stability of the positive associations between AGP concentrations and both depressive disorder and their symptom severity, as detailed in Table S4. Furthermore, to examine the relationship of AGP concentrations with depressive disorder in the unweighted data, we also use the multivariate logistical and linear models to assess the positive relationship between AGP concentrations and depressive disorder and PHQ-9 values and the associations were stable (Table S5). Emerging evidence suggests that autoimmune disorders, including Hashimoto’s thyroiditis and rheumatoid arthritis, may modulate AGP concentrations, potentially confounding the observed association between AGP and depression [40, 41]. To address this potential bias, we additionally adjusted for these autoimmune conditions in our weighted multivariable logistic and linear regression models to control for their potential confounding effects on the relationship of AGP with depression, and a positive relationship remained robust in Table S6.

## Discussion

This cross-sectional investigation utilized nationally representative data from the 2021–2023 cycle of NHANES to evaluate the relationship between AGP concentrations and depressive disorders. Analytical models incorporating sampling weights - including multivariable-adjusted logistic and linear regression models, and RCS models - consistently demonstrated significant positive linear associations with both depression and continuous PHQ-9 scores. In addition, subgroup analyses confirmed this association’s robustness across key demographic and clinical subgroups. Sensitivity analyses incorporating multiple imputations for missing data and alternative model specifications further validated these findings, suggesting serum AGP may serve as a promising inflammatory marker for depression in clinical practice.

AGP, a sensitive acute-phase inflammatory marker, demonstrates rapid elevation during early inflammatory responses, serving as a reliable indicator of systemic inflammation [42]. While emerging evidence suggests a potential association between AGP and depression, current findings remain inconsistent due to limited studies. In this cross-sectional study, our findings showed that AGP concentrations were linked with depressive disorders, which was consistent with Bahrini et al.‘s study in which the relationship of AGP concentrations with depressive symptoms was explored in the Middle Eastern populations [27]. However, our results differed from those of Zarate-Ortiz et al.‘s study [28], which revealed that the relationship between them was not statistically significant. The reason might be that the sample size was insufficient, which could not represent the real serum AGP concentrations of the general population, and the AGP detection technology at that time was not perfect, which caused the detected AGP concentrations was deviate from the true AGP concentrations.

Accumulating epidemiological evidence demonstrates a significant positive association between elevated inflammatory markers and depression risk. For example, A multi-wave longitudinal study (*n* = 1207 adults) revealed that low-grade systemic inflammation mediated the association between victimization and depression development [43]. In addition, some evidence also supports that compared to the normal populations, the levels of proinflammatory cytokines such as C-reactive protein, interleukin-6 (IL-6), and tumor necrosis factor-alpha (TNF-a) have been consistently observed in individuals with major depressive disorder compared to healthy controls across multiple cohort studies [44–47]. In 12 longitudinal studies about the relationship of blood cytokines in adolescents with major depressive disorder (MDD), their results consistently revealed that for adolescents with MDD, their peripheral blood cytokines concentrations, such as TNF-α and IL-8 were remarkably increased [48]. In addition, in a cross-sectional and longitudinal study of 884 adults engaged from South Korea, these authors found that serum high-sensitivity C-reactive protein was a biomarker for suicidal behavior in depressive patients, the concentration of which was elevated [49].

Although the precise biological mechanisms linking inflammation and depression remain incompletely elucidated, current evidence suggests several plausible pathways. Experimental studies have demonstrated that proinflammatory cytokines, particularly IL-6 and TNF-α, may contribute to depression pathogenesis through multiple mechanisms: (1) disrupting monoamine neurotransmitter synthesis and metabolism; (2) inducing structural and functional alterations in glutamatergic neurotransmission; and (3) impairing glucocorticoid receptor signaling [50–52]. These inflammatory mediators may additionally modulate neural circuit activity, leading to neurocognitive dysfunction and core depressive symptoms including anhedonia and persistent low mood [53–55]. Emerging research further implicates metabolic dysregulation in this process, with specific glucose and lipid metabolites potentially mediating inflammation-depression associations. For example, indoleacetic acid, an anti-inflammatory metabolite, has been shown to attenuate depressive-like behaviors in zebrafish models by modulating neuroinflammatory responses [56]. Notably, the gut-brain axis may represent another critical pathway, as elevated inflammatory markers have been associated with altered gut microbiota composition (e.g., reduced Bacteroides and Faecalibacterium, increased Prevotella abundance), which may subsequently influence depression risk through microbial metabolite production and immune modulation [57].

Our study had several strengths. Firstly, this study employed a substantially larger, nationally representative sample compared to prior epidemiological studies examining this association, because some analysis methods incorporated sampling weights to account for the complex survey design of NHANES, thereby ensuring nationally representative estimates and enhancing the generalizability of our findings. Secondly, Serum AGP concentrations were quantified using standardized immunoturbidimetric assays performed in certified clinical laboratories following stringent quality control protocols, which were based on the principle of immunological agglutination with a high sensitivity and specificity in detection efficiency. In other words, target antigens and their corresponding antibodies were facilitated in the reaction system, generating measurable light-scattering aggregates for quantitative analysis, which suggested extremely low serum AGP concentrations also could be detected by this measurement method. Therefore, this methodology provides more accurate estimates of AGP concentrations in the general population compared to prior investigations, in which extremely low serum AGP concentrations could not be detected. Finally, potential confounding variables were systematically adjusted for multivariable models, and comprehensive sensitivity analyses were performed to evaluate the robustness of the observed associations under different models.

Our study also had certain limitations. Firstly, since AGP data was presented only in females, the participants included in this study were only females, and there might be a gender bias in the final results. Secondly, as this study employed a cross-sectional design, the causal relationship between AGP concentrations and depressive disorders could not be established. Thirdly, in this study, we set the criteria of exclusion and inclusion for participants based on AGP data in NHANES (2021–2023) and used them to exclude the participants aged less than 20 years and those with missing AGP data and missing some covariates data, which caused a large proportion of participants to be excluded from the final analysis. Therefore, there might be a choice bias in the final results. Finally, the observed association between AGP concentrations and depressive symptoms may be bi-direction, a relationship that cross-sectional designs could not adequately characterize due to: (1) potential mediation through inflammatory and neuroendocrine pathways, and (2) confounding by psychosocial determinants. Future investigations should employ longitudinal cohort studies to establish temporal relationships, complemented by experimental animal models to elucidate underlying biological mechanisms.

## Conclusion

This cross-sectional study demonstrates a significant, dose-dependent association between serum AGP concentrations and depressive disorders among adult women. These findings advance our understanding of inflammatory mechanisms in depression pathogenesis and highlight the potential therapeutic value of anti-inflammatory interventions in depressive disorder management.

## Electronic supplementary material

Below is the link to the electronic supplementary material.

Supplementary Material 1

## Data Availability

Data will be made available on request.
