# Supplementary material for: Association between the serum alpha-1-acid glycoprotein concentrations and depression in US adult women: a cross-sectional study
Source: BMC Psychiatry. 2025 May 15;25:489. doi: 10.1186/s12888-025-06934-w (PMC12079964; doi:10.1186/s12888-025-06934-w)
Supplement: Supplementary file 1 — Supplementary Material 1 [file 12888_2025_6934_MOESM1_ESM.docx]

Table S1. The knots and AIC values of four RCS models in the relationship between AGP and depression

| Knots | AIC | *P*-value |
| --- | --- | --- |
| N=3 | 2451 | *P* < 0.001 |
| N=4 | 2782 | *P* < 0.001 |
| N=5 | 2943 | *P* < 0.001 |
| N=6 | 3089 | *P* < 0.001 |

Table S2. The knots and AIC values of four RCS models in the relationship of AGP and PHQ-9 scores

| Knots | AIC | *P*-value |
| --- | --- | --- |
| N=3 | 2517 | *P* < 0.001 |
| N=4 | 2696 | *P* < 0.001 |
| N=5 | 2802 | *P* < 0.001 |
| N=6 | 3064 | *P* < 0.001 |

A

B


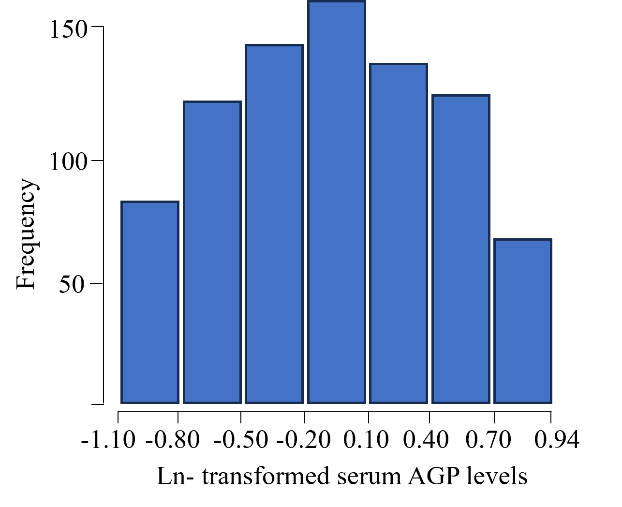

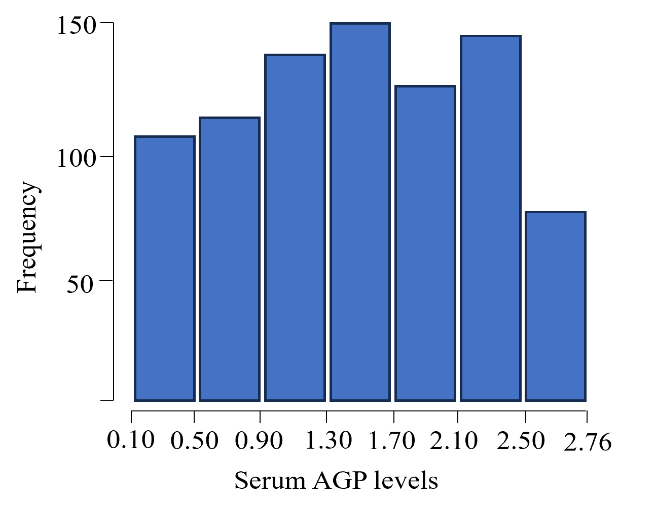


**Fig. S1.** The frequency of serum AGP levels before and after ln-transform. A. The frequency of serum AGP levels. B. The frequency of ln-transformed serum AGP levels.

| Subgroups | *β* (95% CI) | *P*-value | *P* for interaction |
| --- | --- | --- | --- |
| Age  20-34 years  35-49 years | 1.91 (0.46, 3.51)  0.89 (-0.65, 2.52) | 0.008  0.331 | 0.243 |
| Ethnicity  Non-Hispanic White  Non-Hispanic Black  Mexican American  Other races | 2.09 (0.54, 3.59)  -1.38 (-4.13, 1.23)  3.28 (-1.71, 8.98)  0.67 (-1.55, 2.74) | 0.006  0.326  0.196  0.498 | 0.286 |
| Educational levels  Less than High-school  High school  College or above | -1.74 (-8.32, 3.97)  2.23 (-0.85, 5.24)  1.53 (0.31, 2.74) | 0.512  0.184  0.006 | 0.584 |
| Poverty index ratio  PIR<1  1≤PIR<3  PIR＞3 | -3.63 (-7.12, -0.46)  3.72 (1.90, 5.63)  0.87 (-0.36, 2.27) | 0.007  < 0.001  0.265 | 0.275 |
| BMI  Underweight  Normal weight  Overweight  Obesity | 0.86 (0.30, 3.81)  0.60 (-1.29, 2.38)  0.79 (-1.37, 3.16)  1.12 (-1.16, 3.52) | 0.008  0.554  0.501  0.422 | 0.732 |
| Drinking status  Never  Former  Current | 0.39 (-3.89, 4.76)  -0.32 (-4.76, 4.08)  2.14 (0.79, 3.24) | 0.764  0.897  < 0.001 | 0.697 |
| Smoking status  Never  Former  Current | 1.82 (0.62, 3.13)  -1.42 (-4.21, 1.48)  2.21 (-2.14, 6.53) | < 0.001  0.403  0.301 | 0.205 |
| Physical levels  Vigorous  Middle  Other levels | 1.62 (0.17, 2.89)  1.43 (-0.47, 3.72)  0.52 (-3.15, 4.17) | 0.005  0.216  0.725 | 0.712 |
| Hypertension  Yes  No | 0.85 (-1.51, 3.52)  1.82 (0.53, 2.87) | 0.525  0.001 | 0.679 |
| Diabetes  Yes  No | 3.18 (-2.31, 8.83)  1.52 (0.41, 2.63) | 0.264  0.002 | 0.934 |

Table S3. The association of serum AGP levels with PHQ-9 scores

**Notes:** All covariates were adjusted in the models.

Table S4. Association between serum AGP levels and depression and PHQ-9 scores through multiple interpolation methods.

|  | OR (95% CI) | *P*-value | *β* (95% CI) | *P*-value |
| --- | --- | --- | --- | --- |
| Model 1 |  |  |  |  |
| Continuous | 1.91 (1.21, 3.14) | 0.001 | 2.16 (0.99, 3.15) | 0.001 |
| Q1 | Reference (1.00) |  | Reference (0.00) |  |
| Q2 | 1.28 (0.79, 2.23) | 0.278 | 0.63 (-0.39, 1.62) | 0.276 |
| Q3 | 1.51 (0.87, 2.42) | 0.109 | 1.12 (0.13, 2.06) | 0.028 |
| Q4 | 2.15 (1.31, 3.37) | < 0.001 | 1.74 (0.81, 2.63) | < 0.001 |
| *P* for trend | 0.001 |  | 0.001 |  |
| Model 2 |  |  |  |  |
| Continuous | 2.12 (1.33, 3.51) | < 0.001 | 1.73 (0.62, 2.70) | 0.001 |
| Q1 | Reference (1.00) |  | Reference (0.00) |  |
| Q2 | 1.35 (0.81, 2.15) | 0.413 | 0.60 (-0.53, 1.51) | 0.312 |
| Q3 | 1.64 (0.89, 2.27) | 0.264 | 0.89 (-0.04, 1.79) | 0.062 |
| Q4 | 1.86 (1.18, 2.87) | 0.006 | 1.45 (0.52, 2.34) | 0.006 |
| *P* for trend | 0.009 |  | 0.007 |  |
| Model 3 |  |  |  |  |
| Continuous | 1.90 (1.14, 3.25) | 0.007 | 1.35 (0.31, 2.40) | 0.004 |
| Q1 | Reference (1.00) |  | Reference (0.00) |  |
| Q2 | 1.24 (0.69, 2.01) | 0.387 | 0.50 (-0.42, 1.47) | 0.389 |
| Q3 | 1.48 (0.78, 2.18) | 0.413 | 0.81 (-0.25, 1.89) | 0.127 |
| Q4 | 1.69 (1.15, 2.52) | 0.004 | 1.24 (0.28, 2.17) | 0.003 |
| *P* for trend | 0.007 |  | 0.005 |  |

**Notes:** all covariates were adjusted in the models.

Table S5. Association between serum AGP levels and depression and PHQ-9 scores through unweighted multivariate logistic models and unweighted multiple linear models.

|  | OR (95% CI) | *P*-value | *β* (95% CI) | *P*-value |
| --- | --- | --- | --- | --- |
| Model 1 |  |  |  |  |
| Continuous | 3.29 (1.28, 8.04) | < 0.001 | 2.49 (0.68, 4.15) | 0.004 |
| Q1 | Reference (1.00) |  | Reference (0.00) |  |
| Q2 | 1.29 (0.61, 2.06) | 0.387 | 0.69 (-0.35, 1.80) | 0.239 |
| Q3 | 1.58 (0.79, 2.33) | 0.215 | 1.31 (-0.29, 2.34) | 0.187 |
| Q4 | 2.24 (1.32, 3.63) | < 0.001 | 2.14 (1.13, 3.24) | < 0.001 |
| *P* for trend | 0.001 |  | < 0.001 |  |
| Model 2 |  |  |  |  |
| Continuous | 2.91 (1.21, 7.35) | 0.002 | 2.30 (0.38, 4.20) | 0.005 |
| Q1 | Reference (1.00) |  | Reference (0.00) |  |
| Q2 | 1.22 (0.69, 2.21) | 0.326 | 0.73 (-0.31, 1.64) | 0.226 |
| Q3 | 1.48 (0.84, 2.68) | 0.235 | 1.24 (-0.23, 2.24) | 0.143 |
| Q4 | 1.96 (1.28, 3.52) | 0.002 | 1.86 (0.71, 2.73) | 0.002 |
| *P* for trend | 0.008 |  | 0.004 |  |
| Model 3 |  |  |  |  |
| Continuous | 2.73 (1.12, 6.74) | 0.008 | 2.12 (0.20, 4.21) | 0.012 |
| Q1 | Reference (1.00) |  | Reference (0.00) |  |
| Q2 | 1.13 (0.64, 2.06) | 0.453 | 0.52 (-0.32, 1.50) | 0.312 |
| Q3 | 1.32 (0.80, 2.53) | 0.419 | 0.87 (-0.12, 1.96) | 0.087 |
| Q4 | 1.68 (1.14, 2.69) | 0.023 | 1.43 (0.38, 2.42) | 0.007 |
| *P* for trend | 0.029 |  | 0.009 |  |

*Notes:* all covariates were adjusted in the models.

Table S6. Association between serum AGP levels and depression and PHQ-9 scores after additionally adjusting for the covariates of autoimmune diseases (including Hashimoto's thyroiditis and Rheumatoid arthritis)

|  | OR (95% CI) | *P*-value | *β* (95% CI) | *P*-value |
| --- | --- | --- | --- | --- |
| Model 1 |  |  |  |  |
| Continuous | 3.01 (1.23, 7.68) | <0.001 | 2.24 (0.68, 4.01) | 0.009 |
| Q1 | Reference (1.00) |  | Reference (0.00) |  |
| Q2 | 1.24 (0.76, 2.41) | 0.416 | 0.75 (-0.21, 1.58) | 0.215 |
| Q3 | 1.51 (0.89, 2.53) | 0.229 | 1.14 (0.21, 2.05) | 0.019 |
| Q4 | 2.05 (1.15, 3.22) | <0.001 | 1.87 (0.66, 2.98) | <0.001 |
| *P* for trend | 0.004 |  | <0.001 |  |
| Model 2 |  |  |  |  |
| Continuous | 2.72 (1.09, 6.75) | 0.004 | 2.16 (0.42, 3.92) | 0.035 |
| Q1 | Reference (1.00) |  | Reference (0.00) |  |
| Q2 | 1.16 (0.65, 2.23) | 0.389 | 0.67 (-0.38, 1.51) | 0.281 |
| Q3 | 1.38 (0.76, 2.42) | 0.295 | 1.09 (0.06, 2.05) | 0.039 |
| Q4 | 1.82 (1.18, 3.08) | 0.005 | 1.54 (0.68, 2.62) | 0.001 |
| *P* for trend | 0.016 |  | <0.001 |  |
| Model 3 |  |  |  |  |
| Continuous | 2.51 (1.03, 6.24) | 0.031 | 1.87 (0.06, 3.74) | 0.042 |
| Q1 | Reference (1.00) |  | Reference (0.00) |  |
| Q2 | 1.06 (0.52, 1.85) | 0.467 | 0.42 (-0.41,1.23) | 0.428 |
| Q3 | 1.19 (0.68, 2.14) | 0.398 | 0.81 (-0.09, 1.78) | 0.079 |
| Q4 | 1.56 (1.08, 2.89) | 0.040 | 1.22 (0.28, 2.04) | 0.018 |
| *P* for trend | 0.031 |  | 0.009 |  |

*Notes:* all covariates were adjusted in the models.
